# Graves’ Disease-Induced Psychosis Refractory to Intensive Medical Management Requiring Non-voluntary Thyroidectomy for Psychosis Resolution: A Case Report

**DOI:** 10.7759/cureus.51547

**Published:** 2024-01-02

**Authors:** Zachary J Anderson, Sreekant Avula, Ameya Kumar, Deepon Sarkar, Laura LaFave

**Affiliations:** 1 Internal Medicine, Hennepin County Medical Center, Minneapolis, USA; 2 Diabetes, Endocrinology, and Metabolism, University of Minnesota, Minneapolis, USA; 3 Endocrinology and Diabetes, Hennepin County Medical Center, Minneapolis, USA

**Keywords:** plasmapheresis, gland, hormone, thyroid, thyrotoxicosis, psychosis

## Abstract

Graves’ disease is an autoimmune disorder characterized by hyperthyroidism, ophthalmopathy, and dermatopathy. The chief thyroid hormone abnormality is the elevation of thyroid hormone, resulting in an overexcitation of the sympathetic and central nervous systems. Psychosis due to Graves’ disease is rarely the first presenting symptom, but it is an essential complication of those with severe or untreated disease. Most patients respond well to standard medical management for Graves’ disease, although there exists a small subset of people who do not. There are few cases describing patients with psychosis without underlying psychiatric disorders who require intensive care admission and thyroidectomy for necessary management of refractory psychosis secondary to thyrotoxicosis. Here, we present a case of a patient without medical or surgical history who presented with severe psychosis due to untreated Graves’ disease requiring non-voluntary thyroidectomy for definitive management.

## Introduction

The thyroid is a gland that regulates the body's metabolism, growth, and development. It consists of the right and left lobes connected by a median isthmus and is situated anterior to the trachea. The thyroid gland is stimulated by thyrotropin, or thyroid-stimulating hormone (TSH), released from the anterior pituitary. TSH is regulated by thyrotropin-releasing hormone (TRH) from the hypothalamus. This is known as the hypothalamic-pituitary-thyroid axis and is maintained by negative feedback mechanisms. On a cellular level, the thyroid gland consists of parafollicular and follicular cells, with thyroid hormones thyroxine (T4) and triiodothyronine (T3) produced by the latter. Most hormones produced and released by the thyroid gland are in the inactive T4 form, which is then peripherally converted to the active T3 form.

Graves' disease is an autoimmune disorder that can lead to hyperthyroidism, ophthalmopathy, and dermatopathy. This disease occurs primarily in women, with a peak incidence between 40 and 60 years of age [1]. The pathophysiology involves autoantibodies that stimulate the thyroid glands' TSH receptor, inducing the follicular cells' continuous stimulation. This process results in diffuse thyroid hypertrophy and hyperplasia from unregulated production of T4 and T3 with resultant suppressed levels of TSH due to negative feedback. The autoantibodies observed in more than 90% of Graves' disease patients are thyroid-stimulating immunoglobulin (TSI) or thyrotropin receptor antibodies (TRAb) [2].

Patients with Graves' disease will experience enlargement of the thyroid gland and may have compressive symptoms if severe. Increased blood flow through the gland may lead to an audible bruit. Sympathetic neurons activated by elevated thyroid hormones produce systolic hypertension, tachycardia, hyperreflexia, weight loss, heat intolerance, and tremors. Thyroid hormones also overstimulate the central nervous system, causing anxiety, irritability, insomnia, and mood lability. In severe cases, patients may have psychosis, which can mimic psychiatric illness or intoxication. Typical medical management consists of oral medications such as propylthiouracil, methimazole, propranolol, and atenolol. In severe cases, such as those with acute cardiomyopathy or thyroid storm, intravenous corticosteroids or plasmapheresis is required to reduce circulating thyroid hormone levels. Rarely is thyroidectomy required for psychosis refractory to medical intervention.

## Case presentation

Our patient is a 54-year-old female with no past medical or psychiatric history or any family history of psychiatric illnesses who was brought into the emergency department by community responders for disorganized behavior in public. She reported to emergency room staff that she was hearing the voice of Jesus telling her to enter a stranger’s home and bring groceries to an acquaintance.

The patient was evaluated by psychiatry in the emergency department. She was afebrile with a blood pressure of 125/63 mmHg and a pulse of 106/min. On mental status examination, she was poorly groomed, upset, guarded, and displayed paranoid ideation (hearing the voice of Jesus telling her to enter a stranger’s home and bring groceries to an acquaintance) with poor judgment and insight. The patient was diagnosed with psychosis and was placed on a 72-hour hold. She was initially treated with antipsychotics (Quetiapine 50 mg BID) and benzodiazepines (Lorazepam 2 mg every two hours as needed)

Over the next five days, the patient refused laboratory testing, and there was no change in her mental status. She was uncooperative with medical staff, often arguing and screaming without violent intent. The patient agreed to a single blood draw on hospital day 5. Her TSH was checked and found to be undetectably low at <0.10 mIU/L (reference: 0.27-4.20 mIU/L). Her free T4 was undetectably high at >7.7 ng/dl (reference: 0.8-1.6 ng/dL), and her total T3 was also undetectably high at >651 ng/dl (reference: 80-200 ng/dL). Endocrinology was consulted while the patient was in the psychiatric ward for concerns about thyrotoxicosis.

Further evaluation revealed an elevated TSI of 25.10 IU/L (reference: <= 0.54 IU/L) and an elevated thyroid peroxidase antibody (TPO) of 52.3 IU/mL (reference: 0.0-9.0 IU/mL) (Table 1).

**Table 1:** Initial lab values TSH: Thyroid stimulating hormone; TSI: thyroid stimulating immunoglobulin; TPO antibody: thyroid peroxidase antibody

Lab Test	Results	References
TSH	<0.10 mIU/L	0.27 – 4.20 mIU/L
FreeT4	>7.7 ng/dl	0.8 – 1.6 ng/dl
Total T3	>651 ng/dl	80 – 200 ng/dl
TSI	25.10 IU/L	= 0.54 IU/L
TPO antibody	52.3 IU/mL	0.0 – 9.0 IU/mL

Collateral information provided by her emergency contact did not note any history of substance use or psychiatric illness but did describe an insidious onset of increasingly strange behavior over the past several months. Given the duration of her altered mental status and the collateral information available, the diagnosis was suspicious for psychosis due to Graves’ disease. The patient continued to refuse to be examined or to initiate medical therapy and was determined not to have decision-making capacity. With ongoing concerns for thyrotoxicosis and potential life-threatening consequences of continuing to remain untreated, she was moved to the Medical Intensive Care Unit (MICU), where she was sedated and intubated for deeper sedation and airway protection. Additional studies, including syphilis serology, ammonia, and vitamin deficiencies, were nonreactive or within normal limits. Complete blood count, basic metabolic panel, urine toxicology screen, and hepatic panel were grossly unremarkable. A CT scan of the head showed no intracranial pathology. 

**Figure 1:**
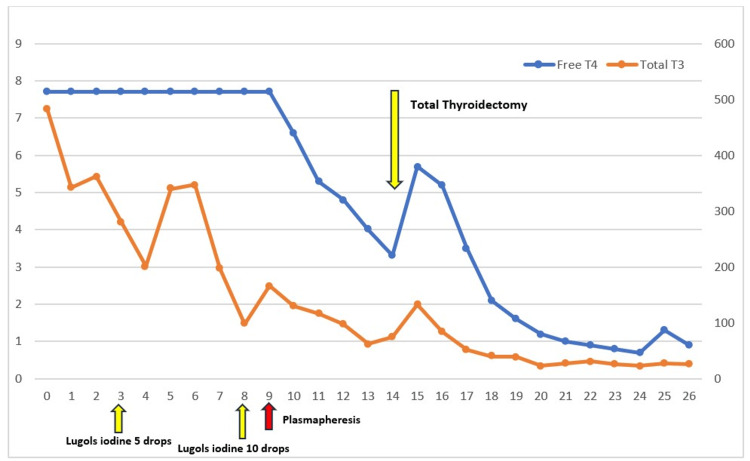
Free T4 levels and total T3 levels as treatment options were implemented. Day 0 is prior to moving into the intensive care unit

In the MICU, we started the patient on medical therapy and began trending free T4 and total T3 throughout her hospitalization (Figure 1).

We began therapy with propylthiouracil 200 mg every four hours and propranolol 60 mg every six hours (treatment day 1). On treatment day 5, the patient’s mental status failed to improve, and she continued to display poor judgment and aggression with staff. We added cholestyramine 4 g twice daily and Lugol’s solution 5% five drops three times a day. By treatment day 7, her behavior had not improved, and her total T3 was still undetectably high. Dexamethasone 3 mg every six hours was added to her treatment plan. Cholestyramine was increased to 4 g four times daily, and Lugol’s solution was increased to 10 drops three times daily on treatment day 8. 

**Figure 2:**
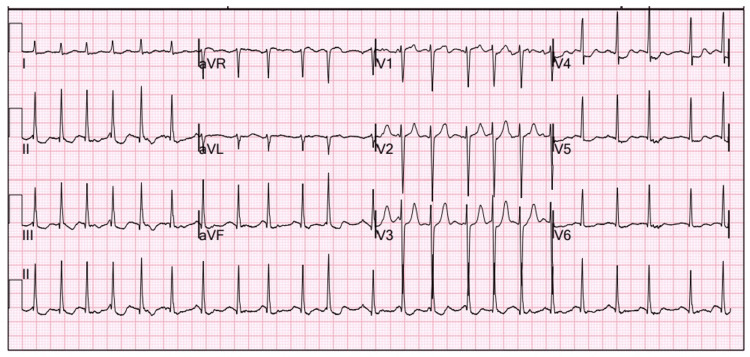
Atrial fibrillation with a rapid ventricular response occurring on treatment day 8

On hospital day 8, the patient developed atrial fibrillation with a rapid ventricular response (Figure 2).

Using the Burch-Wartofsky scale, her score was 55 points (>45 highly suggestive of thyroid storm), suggesting her clinical condition had worsened (Table 2).

**Table 2:** Burch-Wartofsky Point Scale for thyrotoxicosis

Diagnostic Criteria	Scoring
Temperature	
<99 F	0
99 – 99.9 F	+5
100 – 100.9 F	+10
101 – 101.9 F	+15
102-102.9 F	+20
103-103.9 F	+25
>104 F	+30
Central nervous system effects	
Absent	0
Mild (agitation)	+10
Moderate (aggression, psychosis, lethargy)	+20
Severe (coma, seizures)	+30
Gastrointestinal or hepatic dysfunction	
Absent	0
Moderate (pain, diarrhea, nausea)	+10
Severe (jaundice, coagulopathy)	+20
Congestive heart failure	
Absent	0
Mild (edema)	+5
Moderate (rales)	+10
Severe (pulmonary edema)	+15
Atrial fibrillation	
Not present	0
Present	+10
Precipitating event or illness	
No	0
Yes	+10

She earned 10 points for temperature, 30 points for central nervous system effects, 5 points for edema, and 10 points for atrial fibrillation. By treatment day 9, plasmapheresis therapy was initiated, given worsening cardiac symptomatology and continued psychosis with ongoing paranoia and aggression.

**Figure 3:**
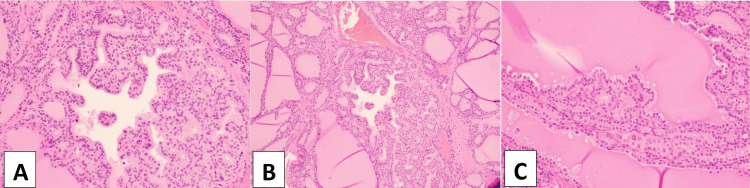
Diffuse hyperplasia and hypertrophy of thyroid tissue. A and B: Hyperplastic follicular epithelium with prominent infolding. C: Pseudopapillary infoldings of hyperplastic columnar follicular cells and colloid with scalloped edges

Atrial fibrillation was difficult to control, requiring titration of metoprolol. On the 11th day of treatment, propranolol was increased to 80 mg every four hours and increased again on the 12th day of treatment to 100 mg every four hours. Given the difficulty in controlling atrial fibrillation and psychosis, with the pharmacological measures already in place, given the severity of thyrotoxicosis, the decision was made to consider the definitive treatment of total thyroidectomy. As the patient was unable to make any decision, the hospital's Ethics Department was consulted. The ethics board made decisions with the involvement of a significant friend and the patient's brother. As there was no improvement in the clinical condition, it was decided to proceed with emergency total thyroidectomy following approval by the Ethics Committee. She underwent a total thyroidectomy on treatment day 14. The thyroid was completely removed without complication, weighing 91.6 g with 9.7 x 7.0 x 3.6 cm measurements. Microscopic evaluation of thyroid tissue was notable for diffuse hyperplasia and hypertrophy, consistent with Graves’ disease (Figure 3).

After the procedure, her thyroid hormone levels began to fall, and there was resolution of her psychosis. There was a gradual improvement in her mental status and resolution of atrial fibrillation. She was transferred out of the intensive care unit, and her mental status was resolving to her pre-morbid state.

## Discussion

Graves’ disease is the most common cause of hyperthyroidism and impacts five times as many women compared to men [3,4]. The classical clinical triad of Graves’ disease involves a diffuse vascular goiter, ophthalmopathy, and pretibial myxedema. There are few case reports of psychosis as the presenting feature of thyrotoxicosis secondary to Graves’ disease, as was the case in our patient. Even rare is the lack of underlying psychiatric disease in our patient, which demonstrates, in this case, the severity of thyrotoxicosis and its effects on the central nervous system. These patients may initially be mistakenly presumed to have a primary psychiatric decompensation, substance use disorder, or symptoms attributed to an underlying psychiatric illness [5,6]. However, ongoing symptoms without resolution or improvement should prompt concern for alternate diagnoses.

In addition to the rare clinical presentation of psychosis due to undiagnosed Graves’ disease, there were many additional challenges with the medical management of her disease. Refusal of medical therapies and lack of capacity necessitated intensive care admission and administration of medications orally through a Corpak and intravenously. Refractory cases of thyrotoxicosis have been reported with Graves’ disease, including thionamides, beta-blockers, steroids, and iodine. However, there are no documented cases of patients with Grave’s disease presenting with psychosis with ongoing aggression, paranoia, and delusions while receiving the standard medical therapies and even daily plasmapheresis. For these reasons, we felt it was in the patient’s best interest to undergo a non-consensual thyroidectomy.

Many other case reports of non-consensual thyroidectomy describe patients with underlying psychiatric illness, which seems to be a risk factor for the development of psychosis in the setting of thyrotoxicosis. Interestingly, our patient had no underlying psychiatric conditions and ultimately did not require antipsychotic therapy following definitive surgical treatment, in contrast to other cases of Graves’ disease-related psychosis [5-10]. Our case demonstrates the rare phenomenon of aggressive and paranoid psychosis from thyrotoxicosis in the absence of underlying psychiatric disease. The anecdotal data from published case reports supports the idea that thyroidectomy is indicated for refractory disease. If the patient lacks capacity, a non-voluntary approach should be taken in the patient’s best interests, consistent with the principle of beneficence [8,9].

Unfortunately, due to a complication from critical illness myopathy, the patient experienced a fatal event before her discharge.

## Conclusions

The correlation between hyperthyroidism and psychosis is intricate and complicated, with underlying mechanisms that are still being investigated. It is essential to identify the psychiatric symptoms of hyperthyroidism promptly to diagnose and intervene in a timely manner. If left untreated, thyroid malfunction can result in significant psychological suffering and impaired functioning. The case we provide illustrates the uncommon occurrence of intense and suspicious mental disorders caused by excessive thyroid hormone levels without any pre-existing psychiatric condition. The empirical evidence from published case reports substantiates the notion that thyroidectomy is warranted for treatment-resistant conditions. If the patient cannot make decisions, a course of action should be pursued without their consent, prioritizing their well-being in line with the concept of doing good. 

## References

[1] Girgis CM, Champion BL, Wall JR (2011). Current concepts in graves' disease. Ther Adv Endocrinol Metab.

[2] Liu T, Zhang X, Long L (2022). Clinical evaluation of an automated TSI bridge immunoassay in the diagnosis of Graves' disease and its relationship to the degree of hyperthyroidism. BMC Endocr Disord.

[3] Kahaly GJ, Grebe SK, Lupo MA, McDonald N, Sipos JA (2011). Graves' disease: diagnostic and therapeutic challenges (multimedia activity). Am J Med.

[4] De Leo S, Lee SY, Braverman LE (2016). Hyperthyroidism. Lancet.

[5] Lazarus A, Jaffe R (1986). Resolution of thyroid-induced schizophreniform disorder following subtotal thyroidectomy: case report. Gen Hosp Psychiatry.

[6] Desai D, Zahedpour Anaraki S, Reddy N, Epstein E, Tabatabaie V (2018). Thyroid storm presenting as psychosis. J Investig Med High Impact Case Rep.

[7] Marian G, Nica A, Ionescu B, Ghinea D (2009). Hyperthyroidism-cause of depression and psychosis: a case report. J Med Life.

[8] Oroko M, Hilmi O, Drummon R (2021). Psychosis and surgery. A case of thyroid storm treated with emergency non-consensual thyroidectomy.. Presented at Society for Endocrinology National Clinical Cases.

[9] Williams OC, Abdulrahim M, Davis V, Jenson C, Anand A, Bachu AK (2022). Management of psychosis associated with Graves' disease: a rare case report. Case Rep Psychiatry.

[10] Rangappa SB, Sharma A, Avula S, Chandramohan D, Sharma V (2023). The intertwined relationship between an overactive thyroid and an overactive mind: a case report and review of literature. Cureus.

